# GNSS/INS Fusion with Virtual Lever-Arm Measurements

**DOI:** 10.3390/s18072228

**Published:** 2018-07-11

**Authors:** Aviram Borko, Itzik Klein, Gilad Even-Tzur

**Affiliations:** Department of Mapping and Geo-Information Engineering, Technion–Israel Institute of Technology, Haifa 3200003, Israel; iklein@technion.ac.il (I.K.); eventzur@technion.ac.il (G.E.-T.)

**Keywords:** inertial navigation system, lever-arm, GNSS

## Abstract

The navigation subsystem in most platforms is based on an inertial navigation system (INS). Regardless of the INS grade, its navigation solution drifts in time. To avoid such a drift, the INS is fused with external sensor measurements such as a global navigation satellite system (GNSS). Recent publications showed that the lever-arm, defined as the relative position between the INS and aiding sensor, has a strong influence on navigation accuracy. Most research in this field is focused on INS/GNSS fusion with GNSS position or velocity updates while considering various maneuvers types. In this paper, we propose to employ virtual lever-arm (VLA) measurements to improve the accuracy and time to convergence of the observable INS error-states. In particular, we show that VLA measurements improve performance even in stationary conditions. In situations when maneuvering helps to improve state observability, VLA measurements manage to gain additional improvement in accuracy. These results are supported by simulation and field experiments with a vehicle mounted with a GNSS and an INS.

## 1. Introduction

Inertial navigation system (INS) and global positioning satellite system (GNSS) fusion aims to utilize the advantages of the two individual systems and overcome their weaknesses. To that end, several coupling architectures for INS/GNSS integration have been proposed. Among them are the loosely coupled (LC) and tightly coupled (TC) approaches [1,2]. In LC integration, both the GNSS and INS operate autonomously to yield their state solution followed by an INS/GNSS integrated solution. Firstly, the raw GNSS measurements are inserted into a standalone GNSS filter to calculate the position and velocity vectors of the GNSS receiver. This GNSS solution is used in aiding the INS filter in a decentralized implementation to estimate the platform states.

In contrast to the LC approach, the TC approach forms an INS/GNSS centralized filter that does not separate the GNSS and INS navigation solutions. The raw GNSS measurements, which are the pseudorange and pseudorange rate, together with those constructed using the INS solution are combined to form the measurements used in the navigation filter [3]. The TC approach provides a more accurate solution than the LC approach as the raw GNSS measurements (pseudorange and pseudorange rate) are introduced directly to the navigation filter [4,5].

In practice, the LC approach [6,7] is more popular since it offers greater flexibility and modularity in terms of system implementation as it allows the use of off-the-shelf hardware that can be easily assembled. In spite of its popularity, the LC approach has a major disadvantage since it requires four or more satellites to form a GNSS position/velocity solution required for the navigation filter. If less than four satellites are available, the navigation solution will rely solely on the standalone INS solution, which, regardless of its grade, drifts in time. There are several solutions to such situations such as the modified loosely coupled approach [8]. This approach makes use of fictitious satellites based on known satellites trajectories and previous vehicle navigation solutions are constructed to enable a pseudo-GNSS solution to aid the INS.

Both LC and TC approaches are widely used in INS/GNSS integrated navigation solutions. As seen in Reference [9], both methods were tested and compared under the goal of achieving continuous and reliable navigation data for vehicles in urban areas. As seen in References [10,11,12], the TC approach was used to enhance ambiguity resolution (AR) for precise GNSS positioning in real time kinematic (RTK) applications, while in References [13,14,15] the TC approach was utilized to obtain a more reliable navigation and attitude determination for lightweight unmanned aerial vehicles (UAVs) using onboard low-cost equipment. In References [16,17], the LC approach was used to enhance georeferencing accuracy for dynamic platforms.

Regardless of the integration approach, the lever-arm between the GNSS receiver (usually mounted on the outside surface of the platform) and INS (usually mounted inside the platform) should be taken into account while calculating the navigation solution. At first, that problem was tackled by considering the lever-arm in the GNSS measurement matrix for position-aided INS [18] or for velocity-aided INS [19]. In that manner, the INS error-state model, commonly the 15 error-state model [7], which is used as the system model for the navigation filter, is left unchanged.

However, due to the locations of the two systems, in practice, it is difficult to accurately measure the lever-arm. In such situations, the lever-arm error will degrade the accuracy of the navigation solution [20,21]. To that end, it was suggested [21] to use the lever-arm error as additional error-states in the navigation filter and thereby increasing the state dimension to 18. Using such an error-state model, an observability analysis of LC INS/GNSS approach was made examining the influence of vehicle maneuvers [22,23].

In this paper, we propose to use a virtual lever-arm (VLA) measurement in addition to a GNSS position or velocity measurements to aid the INS. The motivation for virtual lever-arm measurement is to improve LC approach performance in terms of accuracy and time to converge to steady state solution. The basic idea behind VLA measurement is that, in practice, the lever-arm is known to some accuracy level; however, this knowledge is not utilized directly in the navigation filter, whereas, in some contexts, only to model the stochastic process describing the lever-arm error characteristics. By introducing VLA we utilize this knowledge directly to improve the navigation performance.

The use of virtual measurements, that is translating external knowable on the platform or its operating environment into external measurement to the navigation filter, was proven to be very useful in navigation applications. Nonholonomic constraints in land vehicle navigation [24,25] that translate the fact that the vehicle is travelling on a road and experiences velocity directed only in its longitudinal axis were used as virtual velocity aiding. In addition, zero velocity updates are commonly used in shoe-mounted indoor navigation [26]. At each instance where it is recognized that the foot is resting on the ground, a virtual zero velocity measurement is introduced into the navigation filter.

The VLA measurement is derived in the paper and used to evaluate the INS/GNSS LC navigation filter for different error-state models given the platform dynamics. In particular, we consider here two error-state models: (1) the 18 error-state model to include the lever-arm errors with position measurements; and (2) the 15 error-state model to include the lever-arm states with velocity measurements (the position error-states are not observable and therefore are omitted). A comparison is made between these two models with and without VLA. Both a simulation and a field experiment were used to evaluate the contribution of the virtual lever-arm approach to enhance error-states’ estimation performance.

The rest of the paper is organized as follows: Section 2 presents the navigation equation together with the measurement matrix development for the velocity and the position aided models. Section 3 presents experimental results from numerical simulation tests that examined the VLA contribution to the different models. Section 4 aims to check the findings from the numerical simulation with results from a field test with real data.

## 2. Navigation Equations

In this section the navigation equations of motion, error-state models, and the measurement model used in this paper are introduced.

### 2.1. Kinematic Equations of Motion

The local navigation reference frame was used to represent the position, velocity, and attitude misalignment, while the accelerometer and gyro residuals, modeled as constant bias process, are expressed in the body reference frame. The navigation equations in the navigation frame are [2,7]:

(1) $${\dot{P}}^{n}=V^{n},$$

(2) $${\dot{V}}^{n}=T_{b}^{n}f_{ib}^{b}+g^{n}-\left(\Omega_{en}^{n}+2\Omega_{ie}^{n}\right)V^{n},$$

(3) $${\dot{T}}_{b}^{n}=T_{b}^{n}\Omega_{ib}^{b}-\left(\Omega_{ie}^{n}+\Omega_{en}^{n}\right)T_{b}^{n},$$

where $P^{n}$ and $V^{n}$ are position and velocity vectors expressed in the navigation frame, $g^{n}$ is the gravitation vector, $T_{b}^{n}$ is the transformation matrix from body to navigation frame, $f_{ib}^{b}$ and $\Omega_{ib}^{b}$ are the specific force and skew-symmetric matrix of the angular velocity vector expressed in the body frame, and subscripts ‘i’ and ‘e’ were occasionally used to refer to the inertial frame and the Earth-Centered Earth-Fixed (ECEF) frame, respectively.

### 2.2. Error-State Models

To derive the error-state model, the following error definitions were applied:

(4) $${\hat{P}}^{n}=P^{n}+\delta P^{n},$$

(5) $${\hat{V}}^{n}=V^{n}+\delta V^{n},$$

(6) $${\hat{T}}_{b}^{n}=\left(I+\left[\gamma^{n}\times \right]\right)T_{b}^{n},$$

(7) $${\hat{f}}_{ib}^{b}=f_{ib}^{b}+\varepsilon_{a}+w_{a},$$

(8) $${\hat{\omega }}_{ib}^{b}=\omega_{ib}^{b}+\varepsilon_{g}+w_{g},$$

(9) $${\hat{l}}^{b}=l^{b}+\delta l^{b},$$

where $\delta P^{n}$ and $\delta V^{n}$ are the position and velocity errors vectors, respectively, $\gamma^{n}$ is the attitude misalignment vector and $\left[\gamma^{n}\times \right]$ is the skew-symmetric matrix of $\gamma^{n}$, ${\hat{f}}_{ib}^{b}$ and ${\hat{\omega }}_{ib}^{b}$ are the specific force and gyro measurements, $\varepsilon_{a}$ and $w_{a}$ are the accelerometer bias and zero mean Gaussian white noise, $\varepsilon_{g}$ and $w_{g}$ are the gyro bias and zero mean Gaussian white noise, and $l^{b}$ is the lever-arm vector expressed in the body frame. The system dynamics can be written in matrix form as

(10) $$\delta \dot{x}=A\delta x+Gw,$$

where δx is the error-state vector, A is the system matrix, w is the system noise vector, and *G* is the system noise distribution matrix. In this paper, two error-state models are implemented, one for position aiding and one for velocity aiding as addressed in the following subsections.

#### 2.2.1. 15 Error-State Model

The 15 error-state model was derived to describe a velocity-aided INS with lever-arm vector between the INS and aiding sensor. In this case, the error-state model consists of velocity, attitude, accelerometer, and gyro bias residuals and lever-arm error-states. The position error-states are omitted from the state space model since they are not observable from velocity measurements [27].

When assuming constant biases, the corresponding linear model is given by [1]:

(11) $$\delta {\dot{V}}^{n}=-\left[T_{b}^{n}f_{ib}^{b}\times \right]\gamma^{n}+T_{b}^{n}\varepsilon_{a}+T_{b}^{n}w_{a},$$

(12) $${\dot{\gamma }}^{n}=-T_{b}^{n}\Omega_{ib}^{b}T_{b}^{n}{}^{T}\gamma^{n}+T_{b}^{n}\varepsilon_{g}+T_{b}^{n}w_{g},$$

(13) $${\dot{\varepsilon }}_{a}=0,$$

(14) $${\dot{\varepsilon }}_{g}=0,$$

(15) $$\delta {\dot{l}}^{b}=0.$$

The error-state vector is then

(16) $$\delta x_{15}={\left[\begin{array}{ccc} \delta V & \gamma & \begin{array}{ccc} \varepsilon_{a} & \varepsilon_{g} & \delta l \end{array} \end{array}\right]}^{T},$$

and the corresponding system dynamics and shaping matrix are

(17) $$A_{15}=\left[\begin{array}{ccccc} 0_{3\times 3} & -\left[T_{b}^{n}f_{ib}^{b}\times \right] & T_{b}^{n} & 0_{3\times 3} & 0_{3\times 3}\\ 0_{3\times 3} & -T_{b}^{n}\Omega_{ib}^{b}T_{b}^{n}{}^{T} & 0_{3\times 3} & T_{b}^{n} & 0_{3\times 3}\\ 0_{3\times 3} & 0_{3\times 3} & 0_{3\times 3} & 0_{3\times 3} & 0_{3\times 3}\\ 0_{3\times 3} & 0_{3\times 3} & 0_{3\times 3} & 0_{3\times 3} & 0_{3\times 3}\\ 0_{3\times 3} & 0_{3\times 3} & 0_{3\times 3} & 0_{3\times 3} & 0_{3\times 3} \end{array}\right],$$

(18) $$G_{15}=\left[\begin{array}{c} \begin{array}{cc} T_{b}^{n} & 0_{3\times 3}\\ 0_{3\times 3} & T_{b}^{n}\\ 0_{3\times 3} & 0_{3\times 3} \end{array}\\ \begin{array}{cc} 0_{3\times 3} & 0_{3\times 3}\\ 0_{3\times 3} & 0_{3\times 3} \end{array} \end{array}\right].$$

#### 2.2.2. 18 Error-State Model

The 18 error-state model was derived to describe lever-arm-aided INS with position updates. The 18 error-state model includes all the error-states from the 15 error-state model augmented with the position error-state vector. The model shares the same navigation equations and dynamic linear model as in 15 error-state model (Section 2.2.1), with addition of terms regarding the position error.

The additional position error-states are modeled by

(19) $$\delta {\dot{P}}^{n}=\delta V^{n},$$

the error-state vector, for position-aided INS, is then

(20) $$\delta x_{18}={\left[\begin{array}{cccccc} \delta P & \delta V & \gamma & \varepsilon_{a} & \varepsilon_{g} & \delta l \end{array}\right]}^{T},$$

and the system and shaping matrix are

(21) $$A_{18}=\left[\begin{array}{cccccc} 0_{3\times 3} & I_{3\times 3} & 0_{3\times 3} & 0_{3\times 3} & 0_{3\times 3} & 0_{3\times 3}\\ 0_{3\times 3} & 0_{3\times 3} & -\left[T_{b}^{n}f_{ib}^{b}\times \right] & T_{b}^{n} & 0_{3\times 3} & 0_{3\times 3}\\ 0_{3\times 3} & 0_{3\times 3} & -T_{b}^{n}\Omega_{ib}^{b}T_{b}^{n}{}^{T} & 0_{3\times 3} & T_{b}^{n} & 0_{3\times 3}\\ 0_{3\times 3} & 0_{3\times 3} & 0_{3\times 3} & 0_{3\times 3} & 0_{3\times 3} & 0_{3\times 3}\\ 0_{3\times 3} & 0_{3\times 3} & 0_{3\times 3} & 0_{3\times 3} & 0_{3\times 3} & 0_{3\times 3}\\ 0_{3\times 3} & 0_{3\times 3} & 0_{3\times 3} & 0_{3\times 3} & 0_{3\times 3} & 0_{3\times 3} \end{array}\right],$$

(22) $$G_{18}=\left[\begin{array}{c} \begin{array}{cc} 0_{3\times 3} & 0_{3\times 3} \end{array}\\ \begin{array}{c} \begin{array}{cc} T_{b}^{n} & 0_{3\times 3}\\ 0_{3\times 3} & T_{b}^{n}\\ 0_{3\times 3} & 0_{3\times 3} \end{array}\\ \begin{array}{cc} 0_{3\times 3} & 0_{3\times 3}\\ 0_{3\times 3} & 0_{3\times 3} \end{array} \end{array} \end{array}\right].$$

### 2.3. Measurement Model

**Figure 1:**
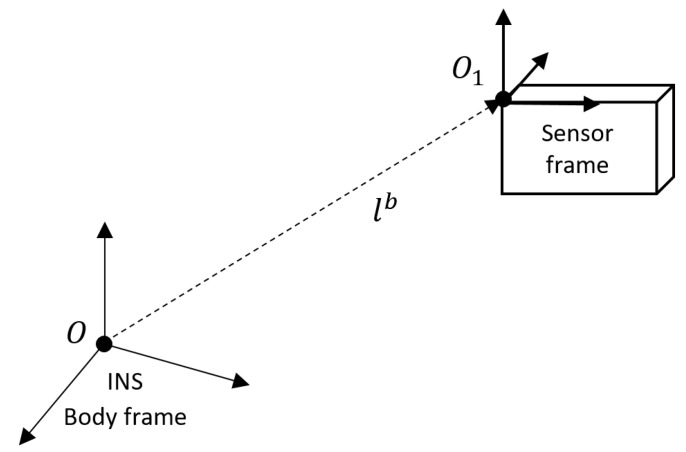
Lever-arm-aided INS with position or velocity measurement sensor (e.g., GNSS antenna).

We consider a typical scenario of aided INS, where a lever-arm vector exists between the two sensors as illustrated in Figure 1. It is assumed that the body reference frame coincides with the inertial sensors sensitive axes and that the lever-arm vector is expressed in the body reference frame. Lever-arm to the measurement sensor may result from the position constraints of the sensor—like open sky for a GNSS antenna. This type of lever-arm may contain values of several meters, especially on large platforms, such as aircrafts or boats, and therefore has a high influence on the navigation solution accuracy [28].

The measurement model can be written as

(23) δz=Hδx+v,

where δz is the measurement’s residual vector, H is the measurement’s design matrix and v is the measurement noise.

In the following sections, this model was developed, and the measurement matrix *H* was described for two measurement aiding sensors—position and velocity. In both aiding methods, the error-state model was augmented with the lever-arm error-states, which reflects that the lever-arm is not considered to be constant. This model is useful if the lever-arm elements are hard to evaluate in a satisfying accuracy and it will be used here for the implementation of the VLA for the performance analysis. The appearance of VLA measurement component in the measurement matrix is shown next.

#### 2.3.1. Velocity Aiding

Consider a velocity measurement with a lever-arm vector relative to INS as shown in Figure 1, were $l^{b}$ is the lever-arm. These velocity measurements can be modeled as [28]:

(24) $$V_{1}^{n}=V^{n}+T_{b}^{n}\left[\omega_{ib}^{b}\times \right]l^{b},$$

where $V_{1}^{n}$ is the velocity measurement vector containing a lever-arm. Let the measurement residual, the difference between the INS-based velocity and measured velocity, be

(25) $$\delta V_{1}^{n}={\hat{V}}_{1}^{n}-V_{1}^{n}.$$

Then, combining Equations (5)–(9) and Equation (24), we obtain

(26) $$\delta V_{1}^{n}=\delta V^{n}+\left(I+\left[\gamma^{n}\times \right]\right)T_{b}^{n}\left(\omega_{ib}^{b}+\varepsilon_{g}\right)\times \left(l^{b}+\delta l^{b}\right)-T_{b}^{n}\omega_{ib}^{b}\times l^{b}.$$

After rearranging and eliminating 2nd order error elements, Equation (26) reduces to

(27) $$\delta V_{1}^{n}=\delta V^{n}-\left[T_{b}^{n}\left(\omega_{ib}^{b}\times \right)l^{b}\times \right]\gamma^{n}-T_{b}^{n}\left[l^{b}\times \right]\varepsilon_{g}+T_{b}^{n}\omega_{ib}^{b}\times \delta l^{b}.$$

Let $L^{b}=\left[l^{b}\times \right]$ and $\Omega_{ib}^{b}=\left[\omega_{ib}^{b}\times \right]$, so that the velocity measurement estimation error can be written as:

(28) $$\delta V_{1}^{n}=\delta V^{n}-\left[T_{b}^{n}\Omega_{ib}^{b}l^{b}\times \right]\gamma^{n}-T_{b}^{n}L^{b}\varepsilon_{g}+T_{b}^{n}\Omega_{ib}^{b}\delta l^{b}.$$

Thus, the measurement matrix for a 15 error-state vector velocity-aided INS in Equation (23) is

(29) $$H_{15}=\left[\begin{array}{cc} \begin{array}{ccc} I_{3\times 3} & -\left(T_{b}^{n}\Omega_{ib}^{b}l^{b}\times \right) & 0_{3\times 3} \end{array} & \begin{array}{cc} -T_{b}^{n}L^{b} & T_{b}^{n}\Omega_{ib}^{b} \end{array} \end{array}\right].$$

#### 2.3.2. Position Aiding

Herein, instead of velocity aided INS we consider a position measurement with lever-arm-aided INS. Position measurements with lever-arm can be modeled as [21]:

(30) $$P_{1}^{n}=P^{n}+T_{b}^{n}l^{b},$$

where $P_{1}^{n}$ represents the position measurement vector with lever-arm.

Let the measurement residual, the difference between the INS-based position and the measurement position, be

(31) $$\delta P_{1}^{n}={\hat{P}}_{1}^{n}-P_{1}^{n}.$$

Then, using Equations (4)–(9) and Equation (30), we obtain

(32) $$\delta P_{1}^{n}=\delta P^{n}+T_{b}^{n}\delta l^{b}+\left[\gamma^{n}\times \right]T_{b}^{n}{\hat{l}}^{b}-\left[\gamma^{n}\times \right]T_{b}^{n}\delta l^{b}.$$

After rearranging and eliminating 2nd order error elements in Equation (32), the position measurement estimation error can be written as:

(33) $$\delta P_{1}^{n}=\delta P^{n}-\left(T_{b}^{n}{\hat{l}}^{b}x\right)\gamma^{n}+T_{b}^{n}\delta l^{b}.$$

Thus, the measurement matrix for an 18 error-state vector position aided INS in Equation (23) is

(34) $$H_{18}=\left[\begin{array}{ccc} I_{3\times 3} & \begin{array}{ccc} 0_{3\times 3} & -\left(T_{b}^{n}{\hat{l}}^{b}x\right) & 0_{3\times 3} \end{array} & \begin{array}{cc} 0_{3\times 3} & T_{b}^{n} \end{array} \end{array}\right].$$

#### 2.3.3. Virtual Lever-Arm Measurements

In order to control the lever-armed system performance, we propose the VLA measurement approach. In general, the lever-arm is known up to some level of confidence, which usually decreases for bigger platforms. However, in most situations this lever-arm remains fixed. The VLA concept takes advantage of that knowledge and translates it into virtual measurements to the navigation filter.

For example, suppose the true lever-arm vector is one meter in each axis. However, due to mechanical constraints, it can be measured up to an accuracy of one centimeter in each axis. Thus, we can assume a virtual sensor measuring the unknown lever-arm error with an accuracy of centimeter. As we show in the results section, this concept helps to improve the navigation performance.

After explaining the basic idea of VLA, we turn to derive the virtual measurement. The VLA measurement residual can be model as

(35) $$\delta l_{1}^{b}={\hat{l}}^{b}-l^{b},$$

where ${\hat{l}}^{b}$ is the lever-arm virtual measurement vector and $l^{b}$ is the known lever-arm value (such as one meter in our pervious example). Combining Equation (9) with Equation (35) we obtain

(36) $$\delta l_{1}^{b}=\delta l^{b},$$

which results in the following measurement matrix, presented here for the 15 (velocity-aided) and 18 (position-aided) error-state models:

(37) $$H_{15\_VLA}=\left[\begin{array}{cc} \begin{array}{ccc} 0_{3\times 3} & 0_{3\times 3} & 0_{3\times 3} \end{array} & \begin{array}{cc} 0_{3\times 3} & I_{3\times 3} \end{array} \end{array}\right],$$

(38) $$H_{18\_VLA}=\left[\begin{array}{ccc} 0_{3\times 3} & \begin{array}{ccc} 0_{3\times 3} & 0_{3\times 3} & 0_{3\times 3} \end{array} & \begin{array}{cc} 0_{3\times 3} & I_{3\times 3} \end{array} \end{array}\right].$$

Augmenting the VLA measurement matrix with a velocity or position measurement matrix, reflected in Equations (29) and (34), respectively, results in the following measurement matrices for the velocity- and position-aided system with VLA measurements:

(39) $$H_{15\_Combined}=\left[\begin{array}{c} \begin{array}{cc} \begin{array}{ccc} I_{3\times 3} & -\left(T_{b}^{n}\Omega_{ib}^{b}l^{b}\times \right) & 0_{3\times 3} \end{array} & \begin{array}{cc} -T_{b}^{n}L^{b} & T_{b}^{n}\Omega_{ib}^{b} \end{array} \end{array}\\ \begin{array}{cc} \begin{array}{ccc} 0_{3\times 3}\; & 0_{3\times 3}\; & 0_{3\times 3}\; \end{array} & \begin{array}{cc} 0_{3\times 3} & I_{3\times 3} \end{array} \end{array} \end{array}\right],$$

(40) $$H_{18\_Combined}=\left[\begin{array}{ccc} I_{3\times 3} & \begin{array}{ccc} 0_{3\times 3} & \;-\left(T_{b}^{n}{\hat{l}}^{b}x\right)\; & 0_{3\times 3} \end{array} & \begin{array}{cc} 0_{3\times 3}\; & T_{b}^{n} \end{array}\\ 0_{3\times 3} & \begin{array}{ccc} 0_{3\times 3}\; & 0_{3\times 3}\; & 0_{3\times 3} \end{array} & \begin{array}{cc} 0_{3\times 3} & I_{3\times 3} \end{array} \end{array}\right].$$

To summarize, instead of using the classical measurement matrix model for velocity (Equation (29)) and position (Equation (34)) aiding, we propose to use them with augmented VLA measurements as in Equation (39) for velocity aiding and Equation (40) for position aiding.

## 3. Simulation Results

To test the performance of the different models, a comprehensive GNSS/INS numerical simulation was constructed. A low-grade IMU (Inertial Measurement Unit) sensor was simulated with an update rate of 100 Hz. The INS solution was corrected with an EKF (Extended Kalman Filter) at 1 Hz using the velocity or position measurements. All measurements were modeled as zero mean Gaussian white noise. The standard deviation (STD) of the position and velocity measurements noise was set to 1 m and 0.8 m/s, respectively. Lever-arm elements were set to [1 1 1] in meters in the body frame. For the VLA measurements, a noise STD was set to 1 mm. The accelerometer constant bias was set to [0.1 0.1 0.1] with noise STD of 0.01 in m/s^2^. The gyro constant bias was set to [10 10 10] with noise STD of 1 in °/h.

**Figure 2:**
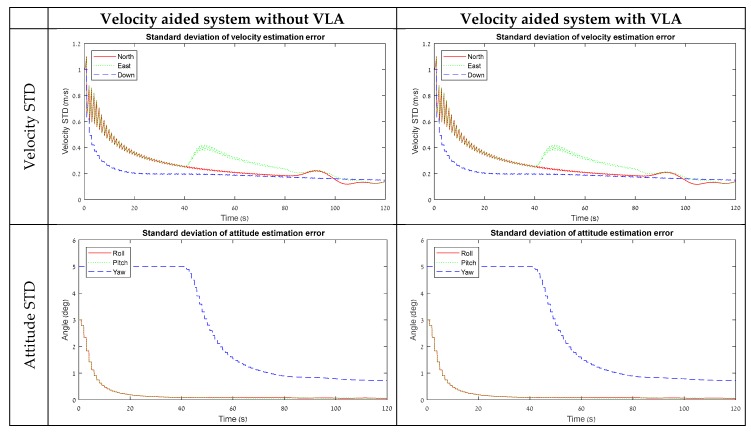

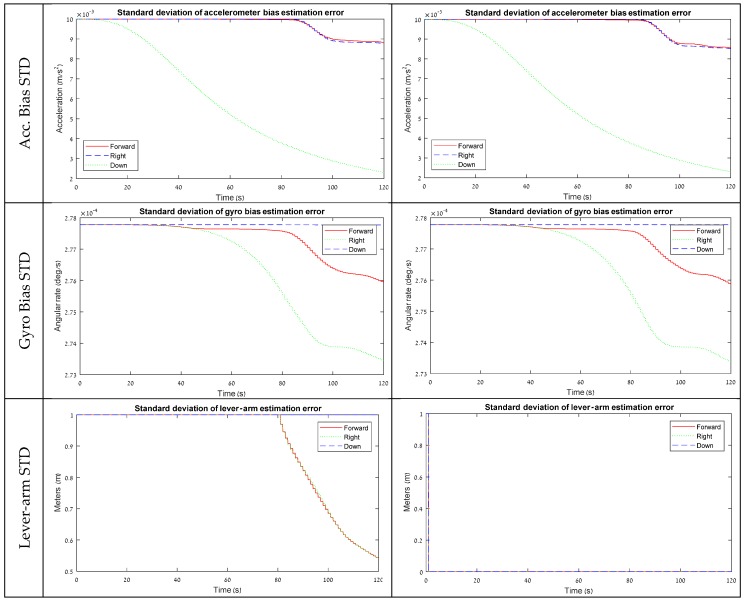
STD results from the software simulation for a velocity-aided system with and without VLA.

**Figure 3:**
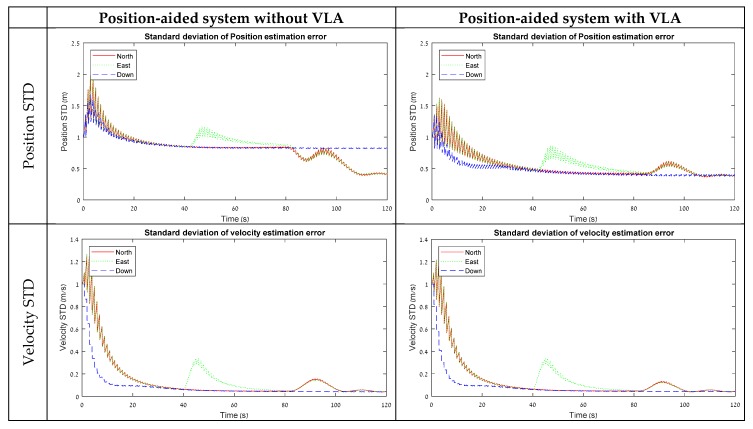

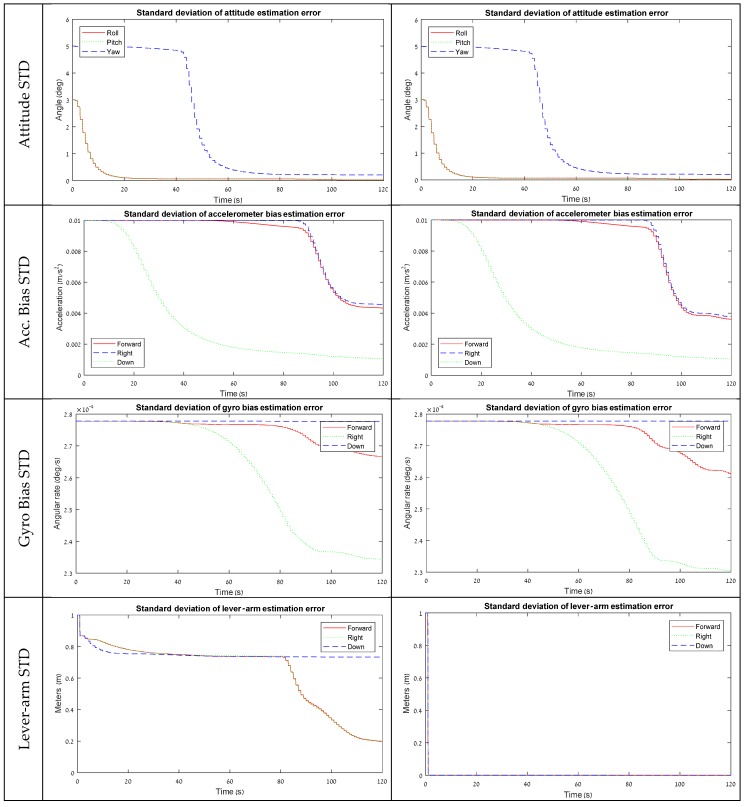
STD results from the software simulation for a position-aided system with and without VLA.

The simulation was implemented for a scenario of 120 s and tested with three types of maneuvers in piecewise linear motion—in the first 40 s the system was in stationary conditions, between 40 to 80 s the system accelerated in X body axis direction, and from 80 to 120 s the system was rotated around the Z axis. The resulting STDs of the error-state covariance for the 15 and 18 error-state models are summarized in Figure 2 and Figure 3, respectively.

### 3.1. Velocity-Aided Model

Figure 2 presents simulation results for a velocity-aided 15 error-state model. According to the lever-arm STD graph, without VLA measurements, the lever-arm elements are not observable for the first 80 s. From second 80, when the system starts to rotate around Z, the horizontal elements of the lever-arm convergent. VLA measurements, on the other hand, make the lever-arm component observable from the beginning. This is an expected result since the lever-arm is measured directly by the VLA measurement. However, the rest of the error-state vector is not affected in a considerable amount. Only a small improvement was detected in the accelerometer and gyro bias STD elements.

This result indicates that the lever-arm elements have a negligible influence on the system state vector when a velocity-aided state model is in use. Thus, for velocity-aided INS, the VLA measurements are not useful for the examined maneuvers.

A possible explanation for this statement can be derived from the observability properties of the lever-arm error-state elements. In Equation (28), these elements appear in the coefficient for the attitude error together with the angular velocity component. As a result, their impact on the estimation performance should be noticed when rotation is applied. Since the lever-arm elements are naturally observable in this case due to the maneuver, as shown, for example, in Figure 2, the contribution of measuring them has a small influence on performance.

### 3.2. Position-Aided Model

Figure 3 shows simulation results for 18 error-state model that is used for position-aided INS. For this model, the lever-arm STD graph without VLA measurements is descending in a fraction from the beginning due to the position measurement, and then remains at the same level during 80 s. Similar to the velocity-aided model, the significant convergence of the lever-arm horizontal elements occurs when the system starts to rotate around Z.

Here, the use of the VLA measurements, as it leads to an improvement in the lever-arm estimation, appears to also affect the performance of the navigation state element estimation. Similar to the velocity-aided model, an improvement was detected in the accelerometer and gyro bias STD elements, but in a more considerable amount. For the position states estimation-wise, the VLA has led to a significant improvement in the STD values in each of the tested maneuvers. A significant improvement was noticed in all three directions when the system was motionless or accelerating in X, and in the ‘Down’ direction when the system was rotating around Z. All of those improvements are related to the unobservable subspaces of the position states with a lever-arm under the different maneuvers, indicating that a VLA measurement improves position state estimation when lever-arm states are not observable in the maneuver.

Contrary to the velocity-aided model, the results here indicate that a position-aided model benefits from estimating the lever-arm elements using the VLA.

## 4. Field Test

**Figure 4:**
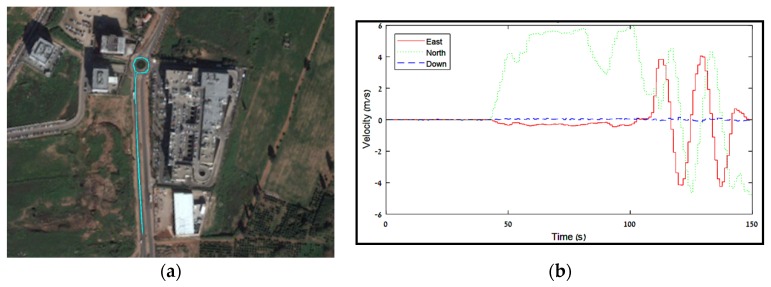
Field test properties. (**a**) Driving path; (**b**) Velocity extracted from the GNSS receiver.

To complete the performance analysis, and to verify simulation findings and conclusions, a field test was conducted. Following the results from the simulation analysis in Section 3, which showed that the lever-arm estimation influence is mostly reflected in the position state estimation for position-aided INS, the field test focused on the position estimation performance with and without VLA. Accelerometer and gyro measurements at 100 Hz from an LG-G3 smartphone were corrected with position information extracted from an external geodetic GNSS receiver (Triumph-1, Javad GNSS Inc., San Jose, CA, USA) with measurements at 1 Hz. Both smartphone and GNSS antenna were installed on top of a vehicle with a lever-arm of [0 1.8 0] meters in the smartphone body reference frame. While measuring, the different maneuvers conducted by the vehicle contain: static condition for 40 s, accelerating and driving in a straight line for 60 s, and, at last, circuited twice in a roundabout. Figure 4 presents the driving path of the vehicle and the velocity information extracted from the GNSS data.

**Figure 5:**
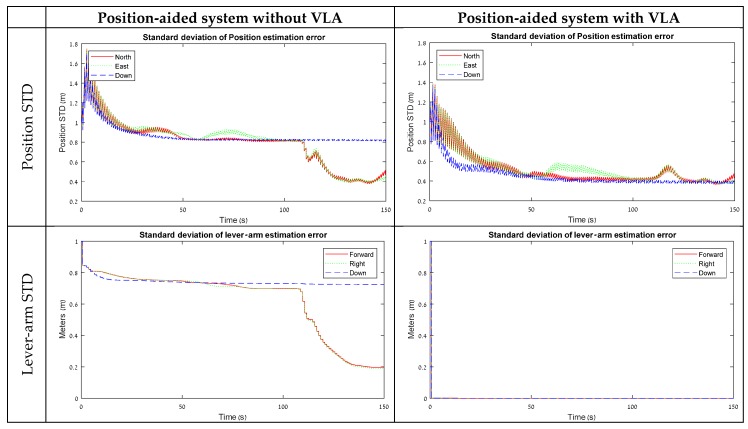
STD results from the field test for position-aided model with and without VLA.

Figure 5 presents the STD results of the position and lever-arm estimation from the field test with the position aiding model. The field experiment results have the same characteristics as the simulation results presented in Section 3.2. Without VLA, the lever-arm horizontal state estimation errors are convergent only when the vehicle starts driving in the roundabout, which can be considered as a rotation around the Z maneuver. The ‘Down’ component of the lever-arm remains on the same level once it stabilizes at the beginning. As a result, the position error-states are stabilized at about 0.8 m during the static and the one-directional motion maneuvers, and, once the vehicle starts its rotation, the horizontal errors drop to 0.4 m, while the ‘Down’ component remains at the same level.

With VLA, on the other hand, as the lever-arm states are artificially measured, all the position states are convergent to a value of 0.4 m from the beginning. This result is adequate with the findings from the simulation and strengthens the statement that VLA measurements contribute to improve navigation performance when lever-arm states are not observable in the maneuver.

## 5. Conclusions

In this paper, VLA measurement was proposed to improve performance of position- or velocity-aided INS. VLA was motivated by the fact that the lever-arm between the GNSS antenna and the IMU’s body frame is usually known for up to some level of confidence and should be reflected in the system to enhance navigation performance.

Numerical simulations and a field experiment with IMU/GNSS have shown that VLA measurements improve the position error-state estimation for position-aided INS. This improvement was especially noticeable in stationary conditions and in other maneuvering types that make the lever-arm elements unobservable. In addition, slight improvements were shown in the accelerometer and gyro bias estimation. On the other hand, VLA measurements did not improve velocity-aided INS and are, therefore, appropriate only for lever-arm position-aided INS.

The simulation and experiment results highlight the VLA contribution for the position-aided INS under representative maneuvering types. According to those results, future implementation of the VLA measurements in navigation applications can benefit from it in terms of the reduced position error, especially on the large-scale platforms where lever-arm components may be difficult to measure and evaluate.
